# Case report: BRCA in the Ashkenazi population: are current testing guidelines too exclusive?

**DOI:** 10.1186/1897-4287-9-3

**Published:** 2011-06-28

**Authors:** Katherine H Saunders, Shivani Nazareth, Peter I Pressman

**Affiliations:** 1 Genetic Risk Assessment Program, Weill Cornell Breast Center, NewYork-Presbyterian Hospital, 425 East 61 Street, 10th Floor, New York, NY 10065, USA

## Abstract

The BRCA1/2 genes account for a significant portion of hereditary breast and ovarian cancers and they are especially prevalent in the Ashkenazi Jewish population. Women who have a mutation can prevent breast and ovarian cancer with surgical intervention. We describe an Ashkenazi Jewish patient who illustrates that current testing criteria are too restrictive, particularly for this population of patients. The patient's sister was diagnosed with breast cancer at age 33; however, she was not a mutation carrier. Based on practice guidelines, the patient was not recommended genetic testing. She subsequently underwent direct-to-consumer (DTC) testing and discovered that she was a mutation carrier. This case demonstrates the need for clinicians to be aware of the higher prevalence of BRCA mutations in the Ashkenazi population. It also exemplifies the need to involve medical professionals, including genetic counselors, in the dissemination of DNA test results.

## 

The BRCA1/2 genes account for a significant portion of hereditary breast and ovarian cancers. BRCA1 mutation carriers have a 47 to 66% chance of developing breast cancer and a 35 to 46% chance of developing ovarian cancer by the age of 70 [1]. BRCA2 mutation carriers have a 40 to 57% chance of developing breast cancer and a 13 to 23% chance of developing ovarian cancer by the age of 70 [1]. These numbers are striking as compared to a 12.5% lifetime risk of breast cancer and a 1.5% lifetime risk of ovarian cancer in the general population.

One out of forty individuals of Ashkenazi Jewish heritage test positive for one of three "founder" mutations: 187delAG and 5385insC in BRCA1 and 6174delT in BRCA2 [2]. This is at least ten times higher than the frequency of mutations in the general population. The importance of identifying carriers is to improve their survival. Women who have a mutation can prevent breast and ovarian cancer with surgical interventions such as bilateral prophylactic mastectomy and/or bilateral prophylactic salpingo-oophorectomy. For those who choose increased surveillance, the aim is to improve survival through earlier discovery and treatment when the cancer is detected. Since eighty-five percent of carriers will develop a breast cancer, in addition to surgical procedures they will usually require systemic therapy, which involves significant expense and side effects, and face the known mortality of the disease. There is no adequate or comparable surveillance for ovarian cancer.

We recently encountered a healthy 30-year-old Ashkenazi Jewish female who did not have a personal history of breast or ovarian cancer. Her family history included a sister who was diagnosed with breast cancer at age 33 [see Figure 1]. The affected sister underwent targeted and complete sequencing for BRCA1 and BRCA2 and no mutation was detected. In adherence with current practice guidelines, our patient had been advised that she did not need to pursue genetic testing. This was because the ideal candidate in the family -- her sister with breast cancer -- had already undergone testing and was not a carrier.

**Figure 1:**
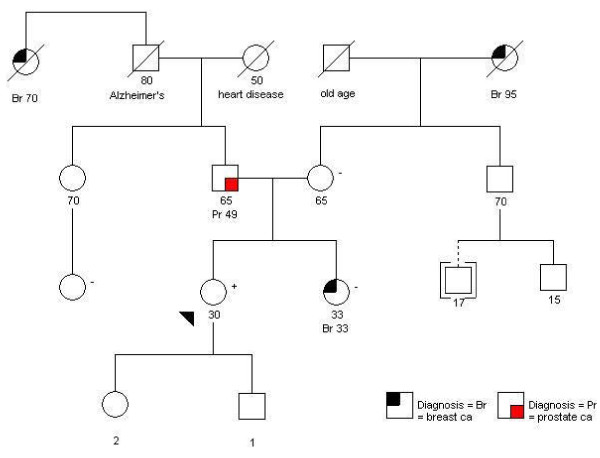
**Patient Pedigree**.

On her own, the patient sent a saliva sample to a direct-to-consumer (DTC) online genetic testing company and discovered that she was a carrier of one of the three Ashkenazi Jewish founder mutations, BRCA1 5385insC. To confirm this result, we submitted a blood sample to Myriad Genetic Laboratory. Since the prevalence of these mutations is high in the Ashkenazi Jewish population, it is not implausible to find a healthy sibling carries a founder mutation, even when the affected sibling is negative. It is also not unusual to detect two different BRCA founder mutations in the same family, often because of masking of transmission through male carriers. It is therefore considered good practice to test all Ashkenazi Jewish candidates for the three founder mutations, even if a mutation has been previously identified in the family.

There has been criticism of DTC marketing; however, this case of serendipitous discovery illustrates a situation where DTC testing was extremely useful. While our patient felt that embarking upon genetic testing over the internet was not ideal, she was grateful to learn about her carrier status. The patient sought genetic counseling and elected to undergo bilateral mastectomies. She has significantly reduced her risk of developing breast cancer, and she also plans to pursue bilateral salpingo-oophorectomy upon completion of childbearing.

Since the three founder mutations account for approximately 96% of mutations in the Ashkenazi population, all such women diagnosed with breast or ovarian cancer are eligible for targeted testing under NCCN guidelines [3]. The founder mutation panel costs $575 through Myriad, versus $3340 for complete gene sequencing. These women need not have any additional family history of cancers. If, however, they are not found to carry a mutation, insurance coverage for other unaffected family members is often limited.

In a recent article by Metcalfe et al., over 2000 Ashkenazi Jewish women were tested for the three founder mutations [4]. Among the mutation carriers, the mean estimate of carrying a BRCA mutation was 3.9%. Only 45% of the carriers, however, met family history criteria for testing. Based on these findings, the authors proposed that general population screening in Ashkenazi Jewish women should be considered. We agree that this would be ideal. Certainly, the criteria for testing should not be as restrictive as currently accepted guidelines. Rubinstein et al. report that over half of unaffected Ashkenazi Jewish BRCA1/2 carriers cannot be identified by relying solely on family history [5]. The authors further conclude that population screening for the founder mutations in the Ashkenazi population is cost-effective. While the financial analysis was based on ovarian cancer outcomes alone, the dollars saved and quality-adjusted life years gained makes the implementation of such a program worthy of further consideration.

This case demonstrates the need for clinicians to be aware of the higher prevalence of BRCA mutations in the Ashkenazi population. The current guidelines do not identify women who may be carriers, to their detriment. Breast cancer is common enough in the Ashkenazi population that random cancers may develop and it should not be assumed that unaffected siblings cannot be carriers. This case also exemplifies the need to involve medical professionals, including genetic counselors, in the dissemination of DNA test results. This patient received notification of her positive carrier status via email format buried amongst other less medically relevant information.

Physicians have learned to follow guidelines in recommending mammography screening for women starting at the age of forty, and earlier for those with a family history of breast cancer. BRCA testing should be incorporated in a similar manner and earlier as the family history dictates. As we approach the era of personalized genomic medicine [6], the role of clinicians will expand to include interpretation of such genetically important findings.

## Consent

Written informed consent was obtained from the patient for publication of this case report and any accompanying images. A copy of the written consent is available for review by the Editor-in-Chief of this journal.

## Competing interests

The authors declare that they have no competing interests.

## Authors' contributions

KS and SN drafted the manuscript and all authors edited and approved the final manuscript.
